# Comparison between Tramadol and Butorphanol for Treating Postoperative Catheter-Related Bladder Discomfort: A Randomized Controlled Trial

**DOI:** 10.1155/2021/6002059

**Published:** 2021-12-28

**Authors:** Feihong Lin, Kaiyang Shao, Wei Pan, Dongdong Liang, Zhangfan Zhao, Jixiang Yuan, Junlu Wang, Ya Lv

**Affiliations:** The First Affiliated Hospital of Wenzhou Medical University, Wenzhou, Zhejiang, China

## Abstract

**Background:**

Intraoperative catheterization often leads to postoperative catheter-related bladder discomfort (CRBD) during the restoration period. This study aimed to assess the curative effect of butorphanol as a K receptor agonist in the treatment of postoperative CRBD. *Patients and Approaches*. Sixty patients with CRBD who underwent elective nonurological surgery at the postanesthesia care unit were randomly and evenly assigned to two groups. The control group was slowly injected with tramadol 1.5 mg/kg using a Murphy dropper, whereas the experimental group was intravenously injected with butorphanol 0.02 mg/kg. Severity, pain score, and sedation score of CRBD were evaluated at 0 min, 5 min, 15 min, 30 min, 1 h, and 6 h later.

**Results:**

The severity score of CRBD and visual analog scale pain score were lower in the butorphanol group than in the control group, whereas the sedation score was higher in the butorphanol group than in the control group.

**Conclusion:**

Butorphanol relieves on postoperative urination discomfort and pain compared with tramadol.

## 1. Introduction

Urethral catheterization is widely adopted during surgeries because it can effectively reduce complications caused by urine retention [1]. Catheter-related bladder discomfort (CRBD) is defined as an uncomfortable feeling in the suprapubic area along with urgent and frequent urination, with or without incontinence [1, 2]. The rate of CRBD can be as high as 47%–90% [2], whereas it is 38%–57% in the postanesthesia care unit (PACU) [3, 4]. Most patients complain moderately about CRBD, which is unendurable and requires therapy [5, 6]. Clinical studies have shown that moderate-to-severe CRBD occurs predominantly in male patients [7], which results in an increased incidence of postoperative agitation [2], extended hospital stay, poor satisfaction, and inevitable financial issues.

The mechanism of CRBD might involve spontaneous contractions of the vesica urinaria with mediation of type 3 hydroxycholine receptor activation [8, 9]. Therefore, muscarinic antagonists have been mainly considered in the prevention and treatment of CRBD in previous studies. Some studies have shown that solifenacin, butylscopolamine, oxybutynin, paracetamol, and tolterodine can improve CRBD symptoms [9–12]. However, these drugs have no satisfactory clinical effects. Therefore, more mechanistic drugs, such as narcotic analgesics, including ketamine, tramadol, dexmedetomidine, lidocaine gel, and dezocine, have attracted the attention of researchers. Antiepileptic drugs, including gabapentin and pregabalin, have been found to prevent or treat CRBD symptoms [1, 13–21]. Despite the availability of these drugs and treatments, the incidence rate of CRBD in existing studies remains as high as 32–69% even after drug intervention, excluding the associated side effects of these drugs [22]. In addition to drug intervention, basal and caudal nerve blocks and dorsal penile nerve block have been proven to have significant therapeutic effects for CRBD [23]; however, nerve damage cannot be completely avoided. Meanwhile, nerve block itself is difficult to operate and cannot be performed if the patient has severe CRBD with restlessness. Therefore, we need to determine a more rapid and safe way to effectively treat or prevent CRBD, to improve patient experience, and to identify medicines to be administered.

Butorphanol is an opioid, a K receptor agonist, and a *μ* receptor antagonist. Κ receptor is an important regulator of visceral pain. After the intravenous injection of butorphanol, the effect starts at 1–5 min (mean 3 min), peaks at 30 min, and has a half-life of 3-4 h. It has the advantages of a strong analgesic effect, long analgesic duration, mild gastrointestinal side effects, less respiratory inhibition, and low drug dependence [24]. In clinical practice, our team has discovered that patients administered butorphanol to manage postoperative pain seemed to have a lower rate of CRBD. At the same time, the butorphanol's effect on the treatment of CRBD has not been reported in recent studies. Therefore, the aim of this study was to assess the curative effects of butorphanol as a K receptor agonist for the treatment of postoperative CRBD.

## 2. Materials and Methods

Our prospective, stochastic, double-blinded study was approved by the Ethics Committee of Wenzhou Medical University (clinical trial number: ChiCTR-INR).

The protocol was approved by the Chinese Clinical Trial Registry (No. ChiCTR2100048644) and was conducted according to the spouse checklist. All patients provided written informed consent before being involved in the study.

### 2.1. Patients

In this study, 66 men with spontaneous CRBD in the PACU were selected, based on the American Society of Anesthesiologists (ASA) physical status ≤2 and CRBD score >2, with 16-French Foley catheters adopted after surgery. We included patients aged 25–84 years who consented to participate in the study. Patients were enrolled in the trial between January 2021 and March 2021. The exclusion criteria were as follows: patients with multiple systemic diseases (cardiovascular, neurological, hepatic, or renal insufficiency), with high blood pressure without control, who were morbidly obese, with mental problems, with past bladder illness or urethral surgery, with bladder or urethral obstruction, with chemical abuse, with chronic pain, those who were unable to communicate efficiently, complicated patients requiring more than two repeated catheters, and patients who were allergic to the drugs in this study.

After the patients were admitted to the operating room and intravenous channels were established, electrocardiogram (ECG) records, peripheral blood oxygen saturation level, blood pressure, and body temperature were monitored for all patients. After 100% oxygen preadministration, anesthesia induction (sufentanil 0.3 *μ*g/kg, propofol 1.5–2.5 mg/kg, and rocuronium 0.6 mg/kg) was performed, and patients were intubated and mechanically ventilated. Subsequently, the 16-French Foley catheter and balloon with 10 mL normal saline were used. The catheter was lubricated with paraffin oil before insertion and fixed to the leg using an adhesive tape with traction after successful insertion. Anesthesia was maintained using a mixture of 2-3% sevoflurane with 50% oxygen and 50% air, with intermittent intravenous injections of sufentanil and rocuronium as needed. After surgery, ondansetron 8 mg was used as a prophylactic drug for postoperative nausea and vomiting. When sufficient natural ventilation was restored and the patient responded to commands, extubation was performed in the PACU. The intraoperative use of drugs that might affect CRBD, such as tolterodine, dexmedetomidine, acetaminophen, tramadol, and butorphanol, was excluded.

The severity of CRBD score was documented as 0, without CRBD complaint even when patients were asked; 1, patients complained only when they were asked; 2, patients complained spontaneously but could tolerate; 3, patients complained spontaneously and they could not tolerate, but lacked behavior-related manifestations; and 4, patients reported spontaneously with behaviors, such as discontent words and seeking to get rid of urinary catheterization.

Postoperative pain was evaluated using the visual analog scale (VAS), with scores ranging from 0 to 10, where 0 represents no pain and 10 represents most severe pain.

Sedative-agitation scores (SASs) were assessed as follows: (1) inability to awaken, no or only slight response to malignant stimuli, and inability to communicate and obey instructions; (2) very calm, responding to physical stimuli, unable to communicate and obey commands, but with voluntary movement; (3) sedative sleepiness and being able to wake up by verbal stimulation or gentle shaking and obey simple commands, but fall asleep immediately; (4) quiet cooperation, quiet easy to wake up, and obey instructions; (5) restlessness, anxiety, or body restlessness, can be calmed by verbal prompt; (6) extremely agitated, requiring protective restraints and repeated verbal cues to dissuade, and biting endotracheal intubation; and (7) dangerous agitation, pulling endotracheal intubation, trying to pull out various catheters, climbing over the window bars, attacking medical staff, and tossing and struggling in bed.

### 2.2. Randomization

Patients who spontaneously complained of CRBD were stochastically divided by the double-blind method into one of the two treatment groups: group A (tramadol, *n* = 30 each) and group B (butorphanol, *n* = 30 each). The assignments came from automatically produced numerical tables that were placed in nontransparent envelopes and opened by two PACU anesthetists administering the medicines in the PACU. The entire results were evaluated by the rest of the anesthetist blinded to the group division. When CRBD (CRBD score >2) was diagnosed in patients in the postanesthetic monitoring treatment room (PACU), patients in group A were treated with 1.5 mg/kg tramadol [22]. Patients in group B intravenously received 0.02 mg/kg butorphanol [25]. All patients were monitored using ECG and by checking levels of peripheral oxygen saturation, blood pressure, and body temperature. CRBD severity, VAS, SAS score, and side effects (nausea, dizziness, headache, and drowsiness) were evaluated at 0 min, 5 min, 15 min, 30 min, 1 h, and 6 h by an anesthetist blinded to the therapy.

The specimen scale was used based on past studies with power analysis [10]. The effect of butorphanol reached its peak 30 min after administration, assuming that the severity of CRBD decreased from 4 to 2 after therapy with butorphanol; 26 patients in each group were required for results to be statistically significant (*a* = 0.05, power = 0.80). To allow for a 10% dropout rate, 30 patients were included per group.

The data obtained in the present study were analyzed using the Statistical Package for Social Sciences version 23 (International Business Machines Corporation, Armonk, New York, USA). The measured data are expressed as mean and standard deviation (*x* ± S). Levene's test was used to test homogeneity of variance, the *W* test was used to test normality, and the paired *t*-test was performed on data conforming to normal distribution and homogeneity of variance. The Mann–Whitney *U* test was used to obtain nonparametric data, and the chi-square test was used for univariate analysis of the groups. The confidence interval was 95%, and a *P* value of <0.05 was considered statistically significant.

## 3. Results

### 3.1. Study Demographics

**Figure 1:**
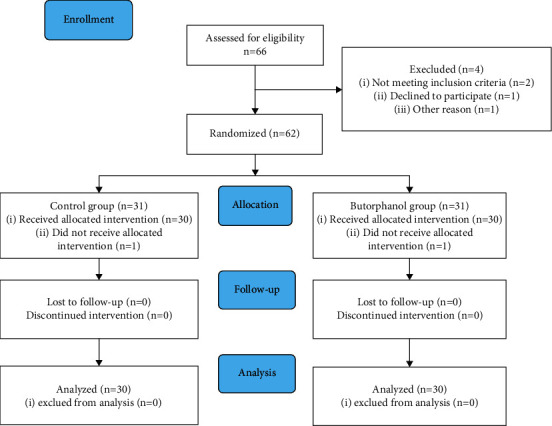
A CONSORT diagram.

A total of 66 male patients with CRBD were screened in this study and four were excluded, of whom one denied participation and one was excluded due to other causes. Sixty-two patients with CRBD were randomly assigned to two groups. One patient from the butorphanol group and one from the control group failed to finish the study as one was transferred to the intensive care unit and one was in a delirious state, respectively. Subsequently, 30 patients from the butorphanol group and 30 patients from the control group were enrolled in the study (Figure 1).

No statistically significant differences existed in height, weight, body mass index (BMI), and ASA classification between the two groups (Table 1). Moreover, there were no statistically significant differences seen in heart rate, mean arterial pressure, and peripheral oxygen saturation level between the two groups at T0–T5 (Table 2).

### 3.2. CRBD Scores

**Figure 2:**
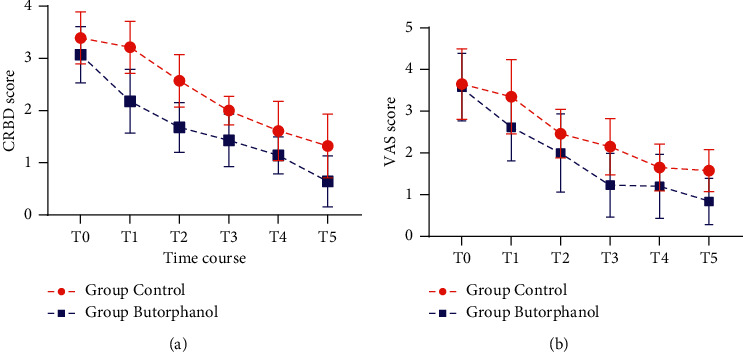
CRBD score (a) and VAS score (b) in butorphanol and control groups at different time points. Time process, T0: 0 min, T1: 5 min, T2: 15 min, T3: 30 min, T4: 1 h, T5: 6 h.

There were significant differences in CRBD severity between the two groups (*P* < 0.05). At 0 min, 5 min, 15 min, 30 min, 1 h, and 6 h after the study drugs were administered, patients in the butorphanol group reported significantly lower CRBD score than those in the control group (Figure 2(a)). We found that butorphanol began to work at 5 min and peaked at 30 min, at which time tramadol began to work. Additionally, butorphanol began to work significantly longer than tramadol.

### 3.3. VAS Scores

Over time, the VAS scores of the control and butorphanol groups showed statistically significant differences (*P* < 0.05). VAS scores of the butorphanol group were lower than those of the control group at 0 min, 5 min, 15 min, 30 min, 1 h, and 6 h after administration (Figure 2(b)).

### 3.4. Adverse Effects between the Two Groups

**Figure 3:**
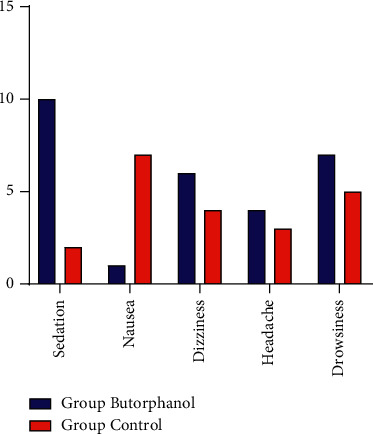
Adverse effects in the two groups (presented as numbers).

The incidence rate of sedation was significantly higher in the butorphanol group than that in the tramadol control group; however, the incidence rate of nausea was significantly higher in the tramadol group than that in the butorphanol group. There were no differences in headache, dizziness, or drowsiness observed (Figure 3).

## 4. Discussion

Inserting an indwelling urethral catheter after the induction of general anesthesia has many advantages, which can avoid psychological tension, discomfort, pain, and embarrassment caused by urethral catheter operation, which is an important measure of nursing. However, with insertion after general anesthesia, the hemodynamic index, degree of agitation, and catheter emergence rate of patients with urethral catheter will increase significantly during the awakening period [26]. As the innervation, such as the parasympathetic and sympathetic nerves, in the urethra, trigone, and bladder neck is rich and the mucous membrane is significantly sensitive to stimuli, inflammatory stimulation or a foreign body in the urethra can easily cause discomfort, such as urgent urination, urination pain, and lower abdominal pain. Urinary catheterization is an invasive procedure and a stressor. Combined with the anatomical characteristics of the male urethra, the discomfort and pain associated with indwelling catheterization are significantly greater than those in women [2]. In addition, the pressure of Foley's catheter bulb on the bladder neck can cause bladder spasm, which will significantly aggravate the patient's symptoms of discomfort, mainly manifested as a sense of urination in the suprapubic area or a sense of urgency and frequency of urination, namely, CRBD [27].

CRBD is commonly observed in the PACU, particularly in male patients. Hence, our team involved male patients for study purposes, and the CRBD rate was 65.2%, which is in accordance with that of a previous study [28]. The high CRBD rate may be associated with the anatomical features, including the long urinary tract and large catheter, as some urology surgeries were performed on the urinary tracts, which remarkably affected our study to diverse degrees. Our study did not involve urology-related patients. In clinical practice, to tackle catheter pain, some teams usually administer lidocaine at the urethral meatus and antalgic medicines, such as fentanyl. Although flurbiprofen axetil can also be adopted, its validity is not satisfactory [29, 30].

CRBD is most likely to cause restlessness in patients during the waking period and is prone to adverse consequences, such as prolapse of endotracheal tubes and drainage tubes, falling from the bed, and incision dehiscence [31]. Clinical studies have shown that tachycardia and elevated blood pressure might lead to cardiovascular and cerebrovascular accidents and endanger life [32, 33]. Based on the results of this study, we also found that most patients experienced restlessness. Postoperative CRBD symptoms [34], such as lower abdomen acid bilges, urethral pain and frequent urination, restlessness, and blood pressure pulse wave, are due to the following four reasons: (1) the bladder neck and triangle area of the bladder are oppressed by Foley's catheter bulb; irritability of nerve endings produces electrical impulses along the lumbar nerve and the spinal cord, and then, these impulses are spread to the brain central sensorium for pain, generating all types of discomfort; (2) the excessive air bag water and pressure on the bladder neck and urethral inner mouth can easily cause bladder spasm, allowing patients feel pain and discomfort, generating a feeling of urination; (3) patients with low pain threshold could not tolerate local stimulation of the urethra by a urethral catheter after an indwelling catheter; and (4) the lack of knowledge on the importance of the indwelling catheter and the way to deal with it can aggravate the discomfort and mental stress for the patient.

**Figure 4:**
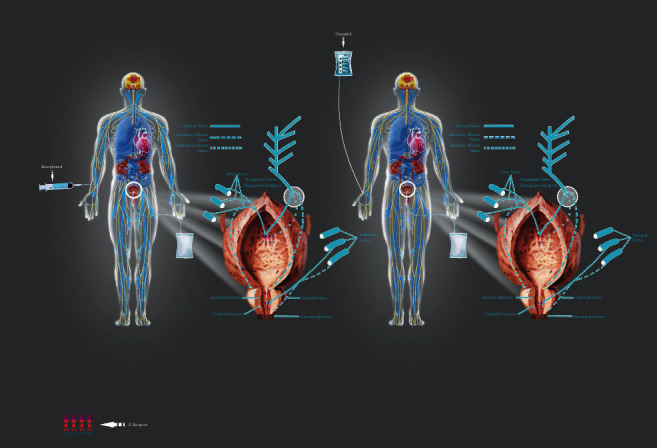
Therapeutic effect of butorphanol on CRBD patients. It is mainly through activating K receptor to inhibit the bladder stimulation into the spinal cord center, thereby improving stress relaxation, relaxation of smooth muscle, and management of visceral pain.

Butorphanol, an opioid, is a K receptor agonist. The K receptor is an important regulator of visceral pain [35]. K receptor agonists can reverse intestinal obstruction caused by acetic peritonitis in rats and block c-Fos expression in the spinal cord, which is also reduced in many upstream brain structures. This shows that it blocks injurious traffic [36]. Butorphanol has the advantages of the strong analgesic effect, long analgesic time, light gastrointestinal side effects, less respiratory inhibition, and low drug dependence. At the same time, butorphanol could significantly prevent postoperative shivering and ease restlessness in patients under general anesthesia [37]. Generally, CRBD is resistant to traditional opioids, but butanediol-activated K receptors might ameliorate CRBD by inhibiting nociceptive stimulation of bladder neck spasm and urinary tract mucosa damage, and butorphanol did not increase the general rate of postoperative queasiness, dizziness, respiratory inhibition, low blood pressure, and hypertension [38]. In contrast, tramadol is a central synthetic opioid analgesic that is routinely used to relieve postoperative pain. Tramadol inhibits M1 and M3 receptors [39], effectively preventing the occurrence and severity of CRBD [40], whereas nausea and vomiting were the common adverse reactions of tramadol [41]. With the score evaluation after butorphanol injection, the patients exhibited a lower CRBD score compared with the tramadol group. Similarly, the evaluation of postoperative pain in the butorphanol group showed a lower score; butorphanol began to work at 5 min and peaked at 30 min, at which time tramadol began to work. Butorphanol works significantly earlier than tramadol, which is the most prominent clinical advantage of butorphanol. Due to the intense nausea and vomiting caused by direct intravenous administration of tramadol, morel titration is more commonly used in clinical use of tramadol, which may itself be the reason for its slower response (Figure 4).

In this study, we also found that two patients were still in a severe CRBD state for a long period after receiving a sufficient amount of experimental drugs; thus, 2 mg/kg propofol injection was administered intravenously to sedate the patients (data are not collected). For patients with severe CRBD, we recommend that propofol be considered an emergency measure.

The current study has some limitations. First, in the general experimental design, although blank control can objectively reflect the efficacy of the drug, the patients selected in this study had CRBD scores of ≥2. If the blank control group is selected, patients in the control group may need to endure certain discomfort for a long period of time and may have self-injury and extreme behaviors of injury and medical care, which is not only not in line with the humanitarian spirit but is also easy to cause hidden dangers of medical safety and increase the consumption of certain medical resources. Second, this study used a single dose of 0.02 mg/kg butorphanol, which has been used to study the efficacy of butorphanol in the treatment of incision pain. We did not evaluate the dose-response effect of butorphanol in the treatment of CRBD, but directly used the measurement of previous studies to conduct a preliminary experiment and then conducted a comparison test between the experimental and control groups after obtaining effective verification. Third, among patients with severe CRBD that could not be alleviated despite the use of the study drugs, if the patients were evidently unable to cooperate with the medical work, we chose to use propofol (2 mg/kg), allowing them to be completely under anesthesia, and then contacted the surgeon to discuss countermeasures. Fourth, CRBD can be caused by a variety of factors, such as foreign body, bladder mucosal injury, operative time, prostate volume, BMI, age, penile block, catheter lubrication, fixation or position of the catheter, and type of postoperative analgesics, and severity and course of CRBD may differ among patients undergoing surgery in different target organs, such as hepatectomy, and these factors have not been further explored. In this study, the incidence and severity of CRBD were evaluated at 0 min, 5 min, 15 min, 30 min, 1 h, and 6 h after surgery. Butorphanol was used to treat CRBD; however, because of the strong sedative effect of butorphanol on patients, some elderly patients were in a state of somnolence after the effect of butorphanol. Thus, the evaluation of the posttreatment CRBD score may not be completely accurate.

## 5. Conclusion

Compared with tramadol therapy at 1.5 mg/kg, butorphanol treatment of 0.02 mg/kg effectively lowered the CRBD score and reduced postoperative pain.

## Figures and Tables

**Table 1:** Descriptive variables for the tramadol control group and butorphanol group.

	Group butorphanol (*n* = 30)	Group control (*n* = 30)	*P* value
Age	58.1 ± 16.5	61.4 ± 12.7	0.380
Weight (kg)	66.0 ± 12.1	64.8 ± 9.4	0.666
Height (cm)	156.8 ± 33.3	166.8 ± 6.0	0.116
BMI (kg/m^2^)	22.3 ± 3.6	23.2 ± 2.6	0.932
Time length of anesthesia (min)	89.1 ± 31.7	80.7 ± 24.4	0.259
ASA (I/II)	21/9	19/11	0.206

**Table 2:** Intraoperative vital signs of patients in both groups.

	Group butorphanol (*n* = 30)			Group control (*n* = 30)		
	HR (bpm)	SPO_2_ (%)	MAP (mmHg)	HR (bpm)	SPO_2_ (%)	MAP (mmHg)
T0	78.3 (13.5)	97.7 (2.4)	113.1 (15.1)	68 (9.8)	99.8 (0.6)	105.5 (10.5)
T1	76.4 (13.7)	97.6 (2.8)	116.1 (17.2)	66 (11.7)	99.8 (0.5)	100.2 (12.1)
T2	75.3 (12.4)	97.8 (2.1)	112.1 (12.1)	65.6 (7.9)	99.8 (0.5)	98.9 (13.2)
T3	76.1 (12.9)	97.3 (2.5)	112.2 (13.6)	65.2 (7.5)	99 (1.3)	98.9 (12.4)
T4	76 (11.2)	97.1 (2.1)	110.9 (12.2)	64.9 (7.5)	98.0 (1.9)	97.8 (11.7)
T5	75.6 (11.4)	97.4 (2.0)	109.9 (12.7)	66.8 (7.7)	98.7 (1.5)	98.6 (10.1)

## Data Availability

Data supporting the findings of this study have not been made available and are available from the corresponding author upon request.

## References

[1] Zhang N., Zhang P., Zhang X., Yang Y. (2012). The efficacy of resiniferatoxin in prevention of catheter related bladder discomfort in patients after TURP - a pilot, randomized, open study. *Translational Andrology and Urology*.

[2] Bai Y., Wang X., Li X. (2015). Management of catheter-related bladder discomfort in patients who underwent elective surgery. *Journal of Endourology*.

[3] Kim H.-C., Lee Y.-H., Jeon Y.-T. (2015). The effect of intraoperative dexmedetomidine on postoperative catheter-related bladder discomfort in patients undergoing transurethral bladder tumour resection. *European Journal of Anaesthesiology*.

[4] Xiaoqiang L., Xuerong Z., Juan L. (2017). Efficacy of pudendal nerve block for alleviation of catheter-related bladder discomfort in male patients undergoing lower urinary tract surgeries. *Medicine*.

[5] Kim H.-C., Park H.-P., Lee J., Jeong M.-H., Lee K.-H. (2017). Sevoflurane vs. propofol in post-operative catheter-related bladder discomfort: a prospective randomized study. *Acta Anaesthesiologica Scandinavica*.

[6] Kunin M., Dinour D., Rosin D. (2018). Intraperitoneal antibiotic administration for prevention of postoperative peritoneal catheter-related infections. *Clinical and Experimental Nephrology*.

[7] Binhas M., Motamed C., Hawajri N., Yiou R., Marty J. (2011). Predictors of catheter-related bladder discomfort in the post-anaesthesia care unit. *Annales Françaises d’Anesthesie et de Reanimation*.

[8] Anderson K. E. (1993). Pharmacology of lower urinary tract smooth muscles and penile erectile tissues. *Pharmacological Reviews*.

[9] Agarwal A., Dhiraaj S., Singhal V., Kapoor R., Tandon M. (2006). Comparison of efficacy of oxybutynin and tolterodine for prevention of catheter related bladder discomfort: a prospective, randomized, placebo-controlled, double-blind study. *British Journal of Anaesthesia*.

[10] Ryu J. H., Hwang J. W., Lee J. W. (2013). Efficacy of butylscopolamine for the treatment of catheter-related bladder discomfort: a prospective, randomized, placebo-controlled, double-blind study. *British Journal of Anaesthesia*.

[11] Srivastava V. K., Nigam R., Agrawal S., Kumar S, Rambhad S, Kanaskar J (2016). Evaluation of the efficacy of solifenacin and darifenacin for prevention of catheter-related bladder discomfort: a prospective, randomized, placebo-controlled, double-blind study. *Minerva Anestesiologica*.

[12] Kim H.-C., Lim S.-M., Seo H., Park H.-P. (2015). Effect of glycopyrrolate versus atropine coadministered with neostigmine for reversal of rocuronium on postoperative catheter-related bladder discomfort in patients undergoing transurethral resection of bladder tumor: a prospective randomized study. *Journal of Anesthesia*.

[13] Safavi M., Honarmand A., Atari M., Chehrodi S., Amoushahi M. (2014). An evaluation of the efficacy of different doses of ketamine for treatment of catheter-related bladder discomfort in patients underwent urologic surgery: a prospective, randomized, placebo-controlled, double-blind study. *Urology Annals*.

[14] Agarwal A., Yadav G., Gupta D., Singh P. K., Singh U. (2008). Evaluation of intra-operative tramadol for prevention of catheter-related bladder discomfort: a prospective, randomized, double-blind study. *British Journal of Anaesthesia*.

[15] Kwon Y., Jang J. S., Hwang S. M., Lee J. J., Tark H. (2018). Intraoperative administration of dexmedetomidine reduced the postoperative catheter-related bladder discomfort and pain in patients undergoing lumbar microdiscectomy. *Journal of Anesthesia*.

[16] Zeeni C., Aouad M. T., Daou D. (2019). The effect of intraoperative dexmedetomidine versus morphine on postoperative morphine requirements after laparoscopic bariatric surgery. *Obesity Surgery*.

[17] Mu L., Geng L.-C., Xu H., Luo M., Geng J.-M., Li L. (2017). Lidocaine-prilocaine cream reduces catheter-related bladder discomfort in male patients during the general anesthesia recovery period. *Medicine*.

[18] Agarwal A., Dhiraaj S., Pawar S., Kapoor R., Gupta D., Singh P. K. (2007). An evaluation of the efficacy of gabapentin for prevention of catheter-related bladder discomfort: a prospective, randomized, placebo-controlled, double-blind study. *Anesthesia & Analgesia*.

[19] Srivastava V. K., Agrawal S., Kadiyala V. N., Ahmed M., Sharma S., Kumar R. (2015). The efficacy of pregabalin for prevention of catheter-related bladder discomfort: a prospective, randomized, placebo-controlled double-blind study. *Journal of Anesthesia*.

[20] Verma R., Agarwal A., Singh P., Gupta D., Shamim R. (2016). Evaluation of efficacy of amikacin for attenuation of catheter-related bladder discomfort in patients undergoing percutaneous nephrolithotomy: a prospective, randomized, placebo-controlled, double-blind study. *Anesthesia: Essays and Researches*.

[21] Ergenoglu P., Akin S., Yalcin Cok O. (2012). Effect of intraoperative paracetamol on catheter-related bladder discomfort: a prospective, randomized, double-blind study. *Current Therapeutic Research*.

[22] Li S., Song L., Ma Y., Lin X. (2018). Tramadol for the treatment of catheter-related bladder discomfort: a randomized controlled trial. *BMC Anesthesiology*.

[23] Li J.-y., Yi M.-l., Liao R. (2016). Dorsal penile nerve block with ropivacaine-reduced postoperative catheter-related bladder discomfort in male patients after emergence of general anesthesia. *Medicine*.

[24] Du B.-X., Song Z.-M., Wang K. (2013). Butorphanol prevents morphine-induced pruritus without increasing pain and other side effects: a systematic review of randomized controlled trials. *Canadian Journal of Anesthesia/Journal canadien d’anesthésie*.

[25] Fu H., Zhong C., Fu Y., Gao Y., Xu X. (2020). Perioperative analgesic effects of preemptive ultrasound-guided rectus sheath block combined with butorphanol or sufentanil for single-incision laparoscopic cholecystectomy: a prospective, randomized, clinical trial. *Journal of Pain Research*.

[26] Srivastava V. K., Agrawal S., Deshmukh S. A., Noushad F., Khan S., Kumar R. (2020). Efficacy of Trospium for prevention of catheter-related bladder discomfort: a prospective, randomized, placebo-controlled, double-blind study. *Korean Journal of Anesthesiology*.

[27] Jang E. B., Hong S. H., Kim K. S. (2020). Catheter-related bladder discomfort: how can we manage it?. *International Neurourology Journal*.

[28] Park J.-Y., Hong J. H., Kim D.-H., Yu J., Hwang J.-H., Kim Y.-K. (2020). Magnesium and bladder discomfort after transurethral resection of bladder tumor. *Anesthesiology*.

[29] Li S. Y., Li H., Ni J., Ma Y. S. (2019). Comparison of intravenous lidocaine and dexmedetomidine infusion for prevention of postoperative catheter-related bladder discomfort: a randomized controlled trial. *BMC Anesthesiology*.

[30] Zhang G.-F., Guo J., Qiu L.-L. (2019). Effects of dezocine for the prevention of postoperative catheter-related bladder discomfort: a prospective randomized trial. *Drug Design, Development and Therapy*.

[31] Yu D., Chai W., Sun X., Yao L. (2010). Emergence agitation in adults: risk factors in 2,000 patients. *Canadian Journal of Anesthesia/Journal canadien d’anesthésie*.

[32] Dahmani S., Stany I., Brasher C. (2010). Pharmacological prevention of sevoflurane- and desflurane-related emergence agitation in children: a meta-analysis of published studies. *British Journal of Anaesthesia*.

[33] Silva L. M. d., Braz L. G., Módolo N. S. P. (2008). Emergence agitation in pediatric anesthesia: current features. *Jornal de Pediatria*.

[34] Viswanathan K., Rosen T., Mulcare M. R. (2015). Emergency department placement and management of indwelling urinary catheters in older adults: knowledge, attitudes, and practice. *Journal of Emergency Nursing*.

[35] Meng J., Jiang S. J., Jiang D., Zhao Y. (2019). Butorphanol attenuates inflammation via targeting NF-*κ*B in septic rats with brain injury. *European Review for Medical and Pharmacological Sciences*.

[36] Bonaz B., Riviere P. J., Sinniger V. (2000). Fedotozine, a kappa-opioid agonist, prevents spinal and supra-spinal Fos expression induced by a noxious visceral stimulus in the rat. *Neuro-Gastroenterology and Motility*.

[37] Manne V. S. K., Gondi S. (2017). Comparison of butorphanol and fentanyl for the relief of postoperative shivering associated with spinal anesthesia. *Anesthesia: Essays and Researches*.

[38] Dinges H.-C., Otto S., Stay D. K. (2019). Side effect rates of opioids in equianalgesic doses via intravenous patient-controlled analgesia. *Anesthesia & Analgesia*.

[39] Bravo L., Mico J. A., Berrocoso E. (2017). Discovery and development of tramadol for the treatment of pain. *Expert Opinion on Drug Discovery*.

[40] Kim D.-H., Park J.-Y., Yu J. (2020). Intravenous lidocaine for the prevention of postoperative catheter-related bladder discomfort in male patients undergoing transurethral resection of bladder tumors: a randomized, double-blind, controlled trial. *Anesthesia & Analgesia*.

[41] Langley P. C., Patkar A. D., Boswell K. A., Benson C. J., Schein J. R. (2010). Adverse event profile of tramadol in recent clinical studies of chronic osteoarthritis pain. *Current Medical Research and Opinion*.

